# Vascular Enlargement as a Predictor of Nodal Involvement in Bladder Cancer

**DOI:** 10.3390/diagnostics13132227

**Published:** 2023-06-30

**Authors:** Alessandra Borgheresi, Andrea Agostini, Francesca Sternardi, Elisa Cesari, Fiammetta Ventura, Letizia Ottaviani, Rocco Francesco Delle Fave, Eugenio Pretore, Alessia Cimadamore, Alessandra Filosa, Andrea Benedetto Galosi, Andrea Giovagnoni

**Affiliations:** 1 Department of Clinical, Special and Dental Sciences, University Politecnica delle Marche, Via Tronto 10, 60126 Ancona, Italy; 2 Department of Radiological Sciences, Azienda Ospedaliero Universitaria delle Marche, Via Conca 71, 60126 Ancona, Italy; 3 Division of Urology, Azienda Ospedaliero Universitaria delle Marche, Via Conca 71, 60126 Ancona, Italy; 4 Division of Pathology, Azienda Ospedaliero Universitaria delle Marche, Via Conca 71, 60126 Ancona, Italy; 5 Department of Biomedical Sciences and Public Healthcare, University Politecnica delle Marche, Via Tronto 10, 60126 Ancona, Italy

**Keywords:** bladder cancer, computed tomography, lymph nodes, staging

## Abstract

In bladder cancer (BC), the evaluation of lymph node (LN) involvement at preoperative imaging lacks specificity. Since neoangiogenesis is paired with lymphatic involvement, this study aims to evaluate the presence of perivesical venous ectasia as an indirect sign of LN involvement, together with other conventional CT findings. All the patients who underwent radical cystectomy (RC) for BC between January 2017 and December 2019 with available preoperative contrast-enhanced CT (CECT) within 1 month before surgery were included. Patients without available pathological reports (and pTNM stage) or who underwent neoadjuvant treatments and palliative RC were excluded. Two readers in blind assessed the nodal shape and hilum, the short axis, and the contrast enhancement of suspicious pelvic LNs, the Largest Venous Diameter (LVD) efferent to the lesion, and the extravesical tumor invasion. In total, 38 patients (33 males) were included: 17 pT2, 17 pT3, 4 pT4; pN+: 20/38. LN short axis > 5 mm, LN enhancement, and LVD > 3 mm were significantly correlated with N+ at pathology. LVD > 3 mm had a significantly higher sensitivity and specificity (≥90%, AUC = 0.949) and was an independent predictor (*p* = 0.0016).

## 1. Introduction

Bladder cancer (BC) is the 10th most common cancer worldwide, with approximately 550,000 new cases annually and estimations in the USA of 82.290 expected cases and 12.160 expected deaths for 2023 [1]. The incidence of BC increases with age (peak 50–70 years), with a male-to-female ratio of 3:1 and a high recurrence rate [2]. In patients with BC, the survival is related to the Tumor, Node, Metastasis (TNM) stage system; the T category is assessed based on the degree of wall invasion, defining T1 as non-muscle-invasive BC (NMIBC) and ≥T2 as muscle-invasive BC (MIBC) [3]. The N stage has a strong prognostic relevance in terms of recurrence-free survival and is related to the T-stage. Lymph node (LN) metastases are rare in NMIBC; they can reach 30% in MIBC and up to 60% when the stage is ≥T3 [4,5,6].

Nodal involvement is assessed on imaging mainly based on LN size, but this criterion alone lacks accuracy since it has been reported that over 90% of normal-sized LNs (short axis ≤ 5 mm) in BC have micrometastases [7]. This is a common issue with other pelvic malignancies, such as rectum [8], prostate and testicles [9], and cervix [10]. The role of Magnetic Resonance Imaging (MRI) in the preoperative evaluation for BC is growing: it is considered superior to CT for T staging because of its higher soft-tissue contrast resolution and the availability of additional functional parameters to improve the specificity in LN evaluation up to 92%; however, its sensitivity is variable across studies (40.7–86%), and MRI is not widely available [7,11,12,13]. Moreover, the reduced spatial resolution of PET limits the assessment of micrometastases in normal-sized LNs (short axis ≤ 5 mm) [14]. Therefore, Computed Tomography (CT) is still one of the main preoperative imaging tools due to its widespread availability [15]; however, the diagnostic accuracy in nodal staging ranges between 54% and 86% in different case series [16,17]. The preoperative nodal understaging at CT in approximately 30% of cases thus leads to the exclusion of the LN size from the AJCC Staging Manual [18,19].

The nodal involvement from MIBC is assessed on the pathological specimen from radical cystectomy (RC) with extended pelvic lymph node dissection, where metastatic LNs are found in 25% of patients [6,20]. Extended pelvic lymph node dissection has demonstrated oncological benefits over less extended dissections, while more aggressive strategies are not beneficial [21]. There is still a debate about the most appropriate template for nodal dissection, the lymphatic drainage of the urinary bladder, and the potential role of sentinel lymph nodes [20,22,23]. The development of a CT imaging biomarker for non-invasive detection of nodal involvement at preoperative imaging would optimize the preoperative staging and allow a less invasive surgery.

In BC, as for other cancers, neoplastic growth is sustained by multiple growth factors, mostly related to neoangiogenesis [24]. The blood vessel density at pathology is related to the metabolic needs of tumoral cells, to the tumor grade, and opens the path for distant dissemination [25]. The increase in lymphatic vessel density follows the expression of lymphangiogenic growth factors [26]; it is involved in the metastatic process together with the increase in vascular density [24,26]. The radiological findings of peritumoral venous invasion in patients with rectal cancer (Rca) and their correlation with nodal and distant metastases have already been explored in rectal cancer (Rca) [27].

This retrospective study aims to correlate the morphological features of LNs together with the extravesical extension of primary tumors and the Largest Venous Diameter (LVD) of the perivesical veins at preoperative CT with nodal involvement at surgical pathology in patients with MIBC.

## 2. Materials and Methods

### 2.1. Study Population

This retrospective study was approved by the local IRB, and the informed consent was waived.

We retrospectively included patients who underwent radical cystectomy with extended lymphadenectomy (pelvic lymphadenectomy of internal–external iliac and obturator stations, extended to common iliac sites) for MIBC between January 2017 and December 2019 at the Department of Urology of the University Hospital “Azienda Ospedaliero Universitaria delle Marche”. All the patients included had a complete pathology report of the surgical specimen (pathological TNM, pTNM) [18,19] and a contrast-enhanced CT urography scan within 1 month before surgery.

Patients without complete clinical or pathological records, without available preoperative contrast-enhanced CT urography, or who underwent neoadjuvant treatments or cystectomies for non-neoplastic diseases were excluded.

### 2.2. Image Acquisition

CT urographies were performed with a 64-row CT scanner (LightSpeed VCT, GE Healthcare, Milwaukee, WI, USA) and a multiphasic protocol with the administration of contrast material (Iopamidol 370 mg [I]/mL, Bracco, Milan, Italy). After a basal acquisition, the post-contrast study was performed with a bolus-tracking technique by placing a region of interest (ROI) in the abdominal aorta and a threshold of 120 HU. The arterial phase was acquired with a delay of 12 s after the threshold, and the venous phase was acquired at 40 s after the arterial phase. The urographic phase was obtained 15 min after the contrast injection. All the CT scans were performed with a tube potential of 120 kV, modulated mA, rotation time of 0.6 s, spiral pitch factor 0.984, and a collimation of 64 × 0.625 mm. Images were reconstructed with a thickness/spacing of 2.5/2.5 mm, Adaptive Statistics Iterative Reconstruction (ASIR, GE Healthcare, level 60%), and a soft kernel. Multiplanar reconstructions on coronal and sagittal planes were obtained.

### 2.3. Image Analysis

The preoperative CT scans were reviewed in separate sessions by two expert radiologists in genitourinary imaging (AA, 10 years, and AB, 8 years of experience) blinded to pathology. The image revision included three categories of findings.

First, the most suspicious LN in terms of dimension (short axis), morphology, or contrast enhancement was evaluated, independently on the side of the primary tumor. On the venous phase of the CT urographies, the following parameters were assessed: shape (oval or round/irregular), presence of fatty hilum, the diameter of the short axis of the biggest LN, and visible contrast enhancement (qualitative assessment: yes/no) [28].

Second, the invasion of perivesical fat was defined as an ill-defined outer layer of the bladder wall, the presence of solid nodules, or perivesical fat stranding [5].

**Figure 1:**
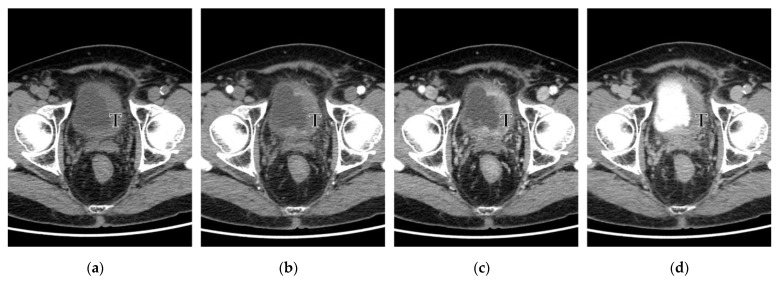

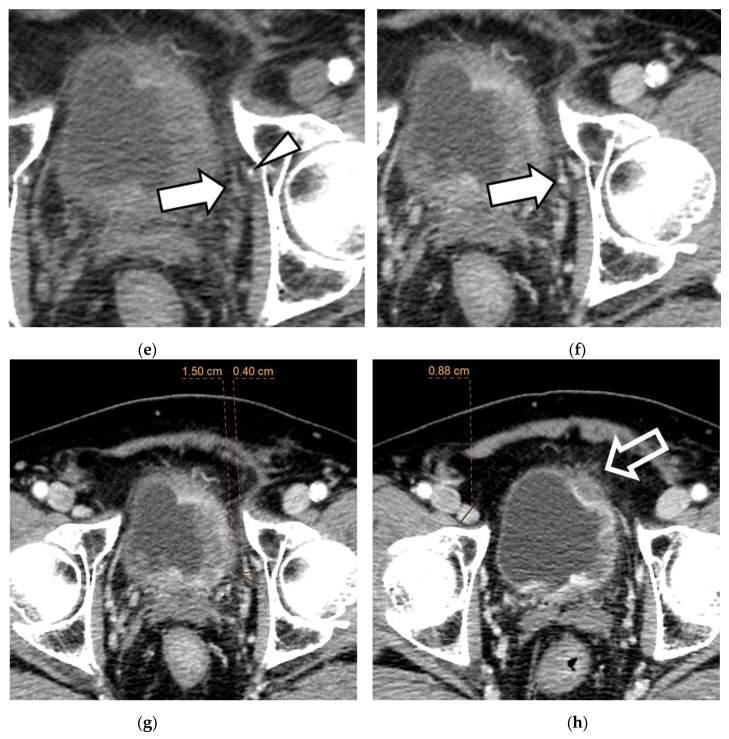
Bladder cancer, stage pT3b pN3. (**a**–**h**): Axial images from CT urography in the basal (**a**), arterial (**b**,**e**), venous (**c**,**f**,**g**,**h**), and urographic phase (**d**). In (**a**–**d**), the primary tumor (T) is highlighted by the letter “T”. (**e**,**f**): Assessment of the perivesical veins. The white arrow points to a perivesical vein (significant enhancement in (**f**), not in (**e**)), while the arrowhead points to an arterial vessel (**e**): the perivesical venous vessel was selected for measurement (white arrow). In **g**, the perivenous vessel shows a diameter of 4 mm and is within the distance of 15 mm from the bladder wall where the primary tumor is located. In a cranial image (**h**), a right iliac LN was measured (short axis of 8.8 mm), while the empty arrow points to perivesical solid tissue with perivesical fat stranding. In this case, the lymphovascular invasion (LVI) was also demonstrated at pathology.

Moreover, as an additional finding, the readers measured the diameter of the largest perivesical venous vessel (LVD) within 15 mm of the bladder wall where the primary tumor was located. In detail, the radiologists assessed the vascular structures in the perivesical fat within 15 mm of the vesical wall, site of the primary tumor, on the pre-contrast, arterial, and venous phase, with the support of multiplanar reconstructions. The vessels with significant arterial enhancement or without any significant enhancement were excluded; the diameter of the largest vessel with venous enhancement was measured (Figure 1).

The discordant qualitative findings were reassessed in consensus by the two readers in a third, separate session one month apart.

### 2.4. Pathology

The pathological TNM stages were extracted from the pathological reports; the lymphovascular invasion (LVI) was also recorded [18,19].

### 2.5. Statistical Analysis

Numerical variables were tested for normality (D’Agostino–Pearson test) and were expressed as the median and interquartile range (25°–75° percentile, 25–75 p). Categorical variables were reported as numbers and percentages.

The average of the quantitative measures of the two readers was used for the statistical analysis. The qualitative and quantitative CT findings were correlated with nodal involvement at pathology (pN+, from the pathological reports) with Receiver Operating Characteristic curves (ROC curves); a cutoff value was obtained for quantitative variables. The comparison of ROC curves was performed. The variables were correlated to pathological nodal status with univariate (Chi-squared test) and multivariate analysis (logistic regression).

The inter-rater agreement was assessed with the Intraclass Correlation Coefficient (ICC).

Statistical analysis was performed using MedCalc v20.218 (MedCalc Software, Ostend, Belgium); *p* values < 0.05 were considered significant.

## 3. Results

### 3.1. Study Population

A total of 120 cystectomies were performed between January 2017 and December 2019. Out of them, five patients were excluded because they underwent cystectomy for non-neoplastic disease (chronic or radiation cystitis). Further patients were excluded: 40 patients had an NMIBC, and 21 patients underwent neoadjuvant treatments. In total, 54 patients underwent radical cystectomy for MIBC; out of them, the preoperative CT was not available in 16 cases.

The final population included 38 patients who underwent radical cystectomy and extended nodal dissection for MIBC, with a median age of 73 y.o. Most of them were males (87%). Table 1 shows the study population characteristics and the stage at pathology as per the *AJCC Staging Manual 8th edition* [19]. All the patients included had a pathological T stage between T2a and T4a, 53% had metastatic pelvic lymph nodes at pathology, and all of them were pM0 (Table 1).

### 3.2. CT Findings Correlated to the Nodal Involvement at Pathology (pN+): ROC Curve Analysis

Table 2 reports the descriptive statistics of the CT findings. The largest pelvic lymph nodes had a median diameter of the short axis of 5.8 mm, an oval shape in 74% of patients, and in 89% of patients the fatty hilum was not visible, while a visible enhancement was detected in 50% of patients. The median LDV was 3.2 mm, and the tumor invasion of the perivesical fat was visible at CT in 13 patients.

The Receiver Operating Characteristic (ROC) curve analysis in Table 3 shows the parameters with the respective thresholds correlated with positive lymph nodes at pathology.

In particular, LNs with a short axis > 5 mm, visible contrast enhancement, and the presence of LVD > 3 mm provided significant results for the prediction of N+ at pathology. Specifically, the nodal short axis > 5 mm had a sensitivity of 80%, a specificity of 72%, and an area under the curve (AUC) of 0.751 (*p* = 0.0021). The visible enhancement of LNs had a sensitivity of 80%, a specificity of 83%, and an AUC of 0.817 (*p* < 0.0001). The LVD > 3 mm achieved a sensitivity of 90% and a specificity of 94% with an AUC of 0.949 (*p* < 0.0001). The other parameters (round/irregular nodal shape, the absence of fatty hilum, and the invasion of perivesical fat) did not provide significant results in the prediction of N+ at pathology (AUC 0.508–0.592, *p* > 0.19, Table 3).

**Figure 2:**
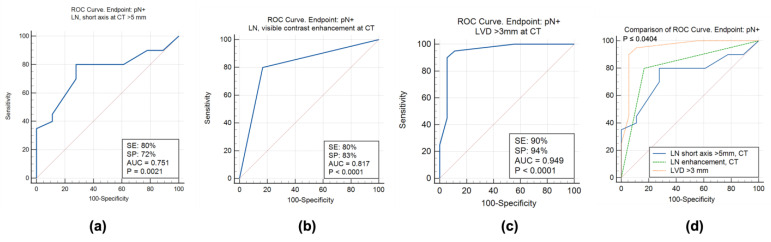
ROC curve analysis. (**a**–**c**) Statistically significant ROC curves for the LN short axis > 5 mm, nodal enhancement, and LVD > 3 mm with pN+ as the endpoint. (**d**) The comparison of ROC curves shows a significantly higher AUC for LVD > 3 mm. LVD: Largest Venous Diameter. SE: sensitivity. SP: specificity. AUC: area under the curve.

Figure 2 shows the statistically significant results of the ROC analysis for the nodal short axis > 5 mm at CT, the visible nodal contrast enhancement at CT, and the LVD > 3 mm (Figure 2a–c). In Figure 2d, LVD > 3 mm had a significantly higher AUC than the visible nodal enhancement (*p* = 0.0404) and the nodal short axis > 5 mm (*p* = 0.0334).

### 3.3. CT Findings Correlated to the Nodal Involvement at Pathology (pN+): Univariate and Multivariate Analysis

**Figure 3:**
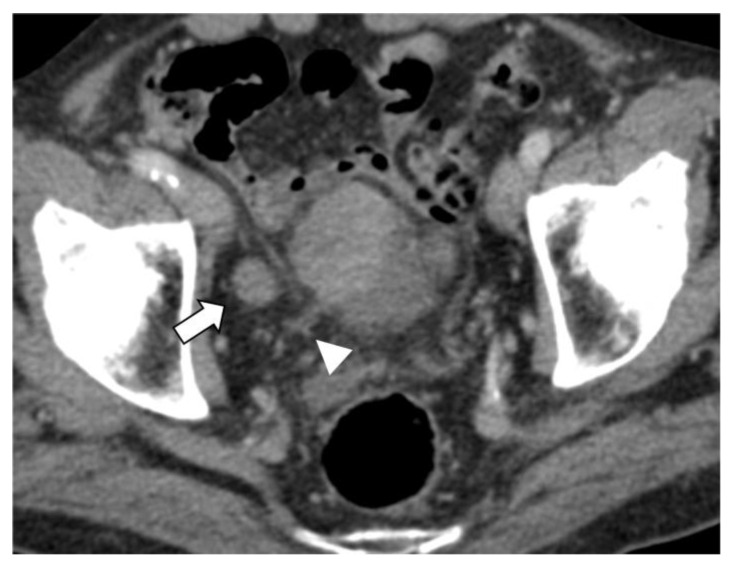
Bladder cancer, stage pT3 pN3. Presence of a metastatic LN with a short axis of 16 mm (arrowhead) and LVD of 5.6 mm (arrowhead) on the axial CT venous phase.

**Figure 4:**
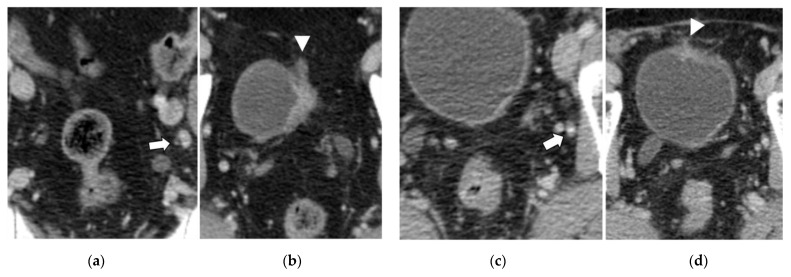
(**a**,**b**): Bladder cancer, stage pT2 pN1. The arrow in (**a**) points to a metastatic LN with visible contrast enhancement and a short axis of 5.6 mm. The arrowhead in (**b**) points to the LVD of 5 mm. (**c**,**d**): Bladder cancer, stage pT2b pN0. In this case, a small, enhancing LN is pointed out by the arrow (short axis 4 mm). In (**d**), the arrowhead points to an LVD of 2 mm, in agreement with the stage pN0.

The univariate analysis with the Chi-squared (χ^2^) test confirmed the results of the ROC analysis, with a significant correlation between nodal short axis > 5 mm, the visible nodal enhancement, and the LVD > 3 mm with N+ at pathology (*p* ≤ 0.0014, Table 4). These three variables were included in the multivariate logistic regression analysis, where the LVD > 3 mm was the only independent predictor of nodal involvement at pathology (OR 26.885, 95% CI: 3.481–107.655, *p* = 0.0016, R2 = 0.830) (Figure 1, Figure 3 and Figure 4).

In addition to the endpoint declared in Table 1 (nodal involvement at pathology), a sub-analysis was performed.

First, the advanced pathological stage T (pT ≥ 3a) was significantly correlated with the nodal involvement at pathology (pN+, χ^2^ = 4.935, *p* = 0.0263). However, when this parameter was included in the logistic regression (endpoint: pN+), it was not retained by the model.

The lymphovascular invasion (LVI) was significantly correlated with the nodal involvement (pN+, χ^2^ = 3.970, *p* = 0.0463) but poorly correlated with the advanced pathological stage T (pT ≥ 3a; χ^2^ = 2.649, *p* = 0.0736). Again, when included in the logistic regression model for the prediction of nodal involvement (pN+), this covariate was not retained.

The LVD > 3 mm was not correlated with the advanced pathological stage T (pT ≥ 3a; χ^2^ = 3.784, *p* = 0.0517). Conversely, the LVD > 3 mm was significantly correlated with the LVI at pathology (χ^2^ = 4.757, *p* = 0.0292).

### 3.4. Inter-Rater Agreement

The inter-rater agreement analysis showed moderate to excellent ICC. In particular, the nodal shape had an ICC of 0.7683 (95% CI: 0.5553–0.8794), the presence of nodal fatty hilum had an ICC of 0.6839 (95% CI: 0.6030–0.7312), the diameter of the short axis had an ICC of 0.9757 (95% CI: 0.9539–0.9873), the assessment of nodal enhancement at CT had an ICC of 0.7990 (95% CI: 0.5455–0.8891), the LVD had an ICC of 0.9595 (95% CI: 0.9190–0.9793), and the tumor invasion of the perivesical fat had an ICC of 0.8650 (95% CI: 0.7417–0.9296).

## 4. Discussion

In this study, we aimed to assess the CT features related to nodal involvement at pathology in patients who underwent radical cystectomy with extended pelvic nodal dissection for MIBC. Three types of CT findings were evaluated: morphological features of suspicious LNs, the tumor invasion of perivesical fat, and the largest diameter of the perivesical veins (LVD).

Regarding the image revision, it must be pointed out that the assessment of the LNs at CT was not related to the side of the tumor. This decision is in line with the available data showing the presence of contralateral nodal metastases in more than 40% of patients with unilateral BC [29,30].

The morphological features of LNs included the diameter of the short axis, the irregular or round shape, the presence of contrast enhancement, and the presence of a visible fatty hilum [28,31]. The data about the normal size of LNs and the correlation between their dimensional increase and metastatic involvement at pathology are not univocal [28]. Vinnicombe et al. [32] published a case series of normal pelvic LNs at CT: the 95th percentile was 6.5 mm in the common iliac stations, 6 mm in the external iliac stations, 5.1 mm in the obturator, and 4 mm in the internal iliac stations. Grubnic et al. [33] published a case series on MRI where the 95th percentile of normal LNs was 4 mm in the common iliac stations, 5 mm in the external iliac stations, 4 mm in the internal iliac, and 5 mm in the obturator stations. In patients with BC, Li et al. [34] found an optimal cut-off of 6.8 mm for the nodal short axis, with a sensitivity of 83%, a specificity of 64.3%, and an AUC of 0.815. Conversely, Caglic et al. [31] suggest a cut-off of 8 mm for the short axis of the positive LN in bladder cancer, confirmed by the European Association of Urology (EAU) [6]. However, Eismann et al. [35] used a cut-off of 15 mm without any significant correlation with the clinical outcomes (cancer-specific survival and overall survival). In the case series of Thoeny et al. [7], 68/88 LNs had a short axis ≤ 3 mm, 13/88 had a short axis > 3 to 5 mm, 5/88 had a short axis > 5 to 8 mm, and 2/88 had a short axis > 8 mm. For these reasons, in our case series, we preferred to calculate the threshold with the ROC analysis rather than to use a defined one. Many of the included patients had relatively small LNs (median diameter 5.8 mm, Table 2), and ROC analysis provided a lower cutoff (5 mm) for suspicious LNs (Table 3). The threshold of 5 mm provided sensitivity, specificity, and AUC values comparable with the literature (80%, 72%, and 0.751, respectively, Table 3 and Table 4, Figure 2). This follows the trend of Li et al. [34].

In agreement with the results of Thoeny et al. [7], we added other morphological features of the LNs, such as the round/irregular shape, the contrast enhancement, and the fatty hilum, as also in Vargas et al. [36]. Interestingly, the qualitative assessment of the contrast enhancement on CT was significantly correlated with the nodal involvement at pathology (80%, 83%, and 0.817, respectively, Table 3 and Table 4, Figure 2), while the shape and the fatty hilum did not. Indeed, in our population, the oval shape and the presence of the fatty hilum were detected in comparable proportions in patients with pN+ and pN−; thus, they were not a significant predictor of nodal involvement [31].

In our results, the morphological features of LN at CT had a trend for diagnostic performance comparable with the literature, with a relatively low sensitivity and a slightly higher specificity, although not significant (Table 3) [37]. All these findings confirm the available evidence about the presence of nodal micrometastases in normal-sized LN and the not relevant morphological changes in metastatic LNs from BC leading to a significant understaging at CT or MRI [5,7,19].

Even though the T stage at pathology is correlated with nodal involvement, in our case series, the tumor invasion of the perivesical fat assessed on CT was not correlated with pN+ (Table 3 and Table 4) [4,5]. Conversely, the advanced pT stage (pT ≥ 3a) and LVI were significantly correlated with the pathological nodal involvement (pN+); however, when introduced into the multivariate model together with the CT parameters, they were not retained [4,5,6,26].

Among the CT features discussed above, only the short axis > 5 mm and the visible contrast enhancement of the LN were included in the multivariate model together with the LVD > 3 mm (Table 4, Figure 2). The presence of an enlarged venous vessel (>3 mm) in the perivesical fat within 15 mm of the primary tumor (LVD) was a strong, independent predictor of nodal involvement (Table 4, Figure 2).

The overexpression of multiple growth factors by BC results in neoangiogenesis and lymphangiogenesis, usually concomitant and crucial for tumor progression, nodal involvement, and metastatic dissemination [24]. Starting from this hypothesis, we attempted to assess the findings on contrast-enhanced CT potentially related to neoangiogenesis, vascular abnormalities, and nodal involvement. Our results demonstrated that the LVD parameter within 15 mm of the bladder wall of the primary tumor (to avoid confusion with other causes of vascular enlargement or congestion [38]) is an independent, strong predictor of the stage pN+, suggesting that abnormal perivesical venous drainage may be related to the nodal dissemination of BC (Table 3 and Table 4, Figure 1, Figure 2, Figure 3 and Figure 4). Moreover, the sub-analysis performed with the Chi-squared test found a significant correlation of LVD > 3 mm with the presence of LVI at pathology. Conversely, the correlation between the LVM > 3 mm and the pT ≥ 3a was not demonstrated.

The correlation between vascular abnormalities, risk of recurrence, nodal and distant metastases, and survival has been demonstrated in rectal cancer (RCa). Krasna et al. [39] found a significantly increased incidence of metastases in the case of venous invasion assessed from pathological specimens of RCa (60% vs. 17%). Subsequently, Bayar et al. [40] performed a revision of the pathological specimens of 59 patients resected for RCa. The authors found that the tumor venous invasion was an independent predictor of nodal metastases with 18-fold increased odds. Katsuno et al. [27] found that circulating free cells in the venous drainage of RCa are strongly related to nodal and hepatic metastases. These results were confirmed by Tsutsuyama et al. [41], further elucidating the relationship between venous drainage and nodal metastases in RCa. These data lead to the definition of extramural vascular invasion (EMVI), described by Smith et al. [42] as the presence of tumor cells within the veins outside the muscularis propria of the bowel wall. In this paper, the authors aimed to create a scoring system for the assessment of EMVI on rectal MRI examinations, and the presence of enlarged veins in the peritumoral mesorectal fat was associated with the highest positive values of the score. More recently, the concept of EMVI in RCa has demonstrated a superior prognostic accuracy compared to the TNM staging system, and it has been incorporated as an independent parameter in the risk assessment of RCa by the European Society for Medical Oncology [43,44].

Historically, the radiological findings of EMVI were assessed on high-resolution MRI, thanks to the better contrast of soft tissues [42]. Initial data are available with CT. Wu et al. [45] found a significant correlation between the increased diameter of the superior hemorrhoidal vein on the preoperative CT of patients with RCa and the presence of lymphovascular invasion at pathology. Similarly, Coruh et al. [46] correlated the diameter of the superior rectal vein–inferior mesenteric vein on preoperative CT with the presence of EMVI at pathology in patients with RCa. The assessment of peritumoral vascular involvement at CT has been performed in anatomical districts that are difficult to evaluate with MRI. Yao et al. [47] assessed the EMVI on CT in patients with colon cancer. The authors demonstrated that CT-EMVI was an independent predictor of disease-free survival [47]. In Cheng et al. [48], the EMVI assessed on CT in patients with gastric cancer was an independent predictor of 1-year progression-free survival together with tumor location and growth pattern.

To our knowledge, this is the first paper to assess the correlation between locoregional venous enlargement and nodal involvement in BC. Despite the small population sample, a correlation between the LVM on CT and the LVI at pathology was also recorded. LVD may become a useful tool to overcome the limitations of CT in the assessment of nodal metastases. In this way, a more accurate nodal assessment may be feasible on preoperative CT, with valuable information about treatment planning to selectively avoid extended lymphadenectomies. Further studies on larger populations are warranted to confirm our results, investigate the underlying pathophysiological mechanisms, demonstrate eventual similarities with the pathophysiological mechanism of EMVI in RCa, and assess the prognostic value.

The present study has several limitations. First, it is a retrospective study on a highly selected, mono-institutional, small cohort. This small population is the result of the strict inclusion criteria, since the patients with NMIBC were excluded; the patients who underwent neoadjuvant treatments were also excluded to avoid potential reactive bladder wall thickening and LN enlargement [49].

## 5. Conclusions

Our results suggest a significant correlation between the nodal short axis > 5 mm, the visible contrast enhancement of LN on CT, and the presence of peritumoral venous enlargement (Largest Venous Diameter, LVD > 3 mm) with the nodal metastases at pathology. At multivariate analysis, the LVD > 3 mm on the venous phase of CT was the only independent predictor of the pN+ stage, suggesting that this finding may be an indirect CT sign of nodal metastases in patients with MIBC. Further studies are warranted.

## Figures and Tables

**Table 1:** Demographics.

Patients (N ≤ 38)	Median (25–75 p) N (%)
Age (y.o.)	73 (68–82)
Gender (M/F)	33 (87%)/5 (13%)
T Stage. Pathology	
pT2a pT2b pT3a pT3b pT4a	6 (15%) 11 (29%) 4 (11%) 13 (34%) 4 (11%)
N Stage. Pathology	
N0 N+	18 (47%) 20 (53%)
M0 Stage. Pathology	38 (100%)
Lymphovascular Invasion (LVI). Pathology	10 (26%)

Legend. 25–75 p: interquartile range. y.o: years old. M: male. F: female.

**Table 2:** CT findings.

CT Finding	Parameter	Median (25–75 p) N (%)
Nodal shape	Round/Irregular	10 (26%)
	Oval	28 (74%)
Fatty hilum	Present	4 (11%)
	Absent	34 (89%)
Nodal short axis (mm)		5.8 (4.0–7.0)
Nodal enhancement	Present	19 (50%)
	Absent	19 (50%)
LVD (mm) *		3.2 (2.5–4.0)
Invasion of perivesical fat	Present	13 (34%)
	Absent	25 (66%)

Legend. 25–75 p: interquartile range. LVD: Largest Venous Diameter. * Within 15 mm of the primary tumor.

**Table 3:** CT findings and ROC curve analysis (Endpoint: pN+).

CT Finding	Parameter/ Cutoff	AUC (95% CI)	Sensitivity (%)	Specificity (%)	*p*
Nodal shape	Round/irregular	0.592 (0.421–0.748)	35.00	83.33	0.1964
Fatty hilum	Absent	0.547 (0.378–0.709)	15.00	94.44	0.3400
Nodal short axis (mm)	>5	0.751 (0.585–0.877)	80.00	72.22	**0.0021**
Nodal enhancement	Present	0.817 (0.658–0.923)	80.00	83.33	**<0.0001**
LVD (mm) *	>3	0.949 (0.824–0.994)	90.00	94.44	**<0.0001**
Invasion of perivesical fat	Present	0.508 (0.341–0.674)	35.00	66.67	0.9161

Legend. 25–75 p: interquartile range. AUC: area under the curve. LVD: Largest Venous Diameter. * Within 15 mm of the primary tumor. The bold highlights the significant results (the other numbers of the column are not significant. It improves the readability of the table.

**Table 4:** Univariate and multivariate analysis of CT parameters correlated to nodal involvement at pathology (pN+).

CT Parameter	Parameter	pN− N (%)	pN+ N (%)	Univariate		Logistic Regression	
				χ^2^	*p*	OR (95% CI)	*p*
Nodal shape	Round/irregular	3 (8%)	7 (18%)	1.599	0.2061	^¥^	
	Oval	15 (40%)	13 (34%)				
Fatty hilum	Present	1 (2%)	3 (8%)	0.874	0.3500	^¥^	
	Absent	17 (45%)	17 (45%)				
Nodal short axis	>5 mm	5 (13%)	16 (42%)	10.175	**0.0014**	^‡^	
	≤5 mm	13 (34%)	4 (11%)				
Nodal Enhancement	Present	3 (8%)	16 (42%)	14.800	**0.0001**	13.208 (0.847–106.049)	0.0656
	Absent	15 (39%)	4 (11%)				
LVD *	>3 mm	1 (3%)	18 (47%)	26.311	**<0.0001**	26.885 (3.481–107.655)	**0.0016**
	≤3 mm	17 (45%)	2 (5%)				
Invasion of perivesical fat	Present	6 (16%)	7 (18%)	0.011	0.9150	^¥^	
	Absent	12 (32%)	13 (34%)				

Legend. χ^2^: Chi-squared test. OR: Odds Ratio. 95%CI: 95% confidence interval. LVD: Largest Venous Diameter. ^¥^: not selected for multivariate analysis. *: within 15 mm of the primary tumor. ^‡^: selected but not retained in the multivariate logistic regression model. The bold highlights the significant results (the other numbers of the column are not significant. It improves the readability of the table.

## Data Availability

The data have been entirely published within the manuscript.
